# Evaluation and Management of Nutritional Consequences of Chronic Liver Diseases

**DOI:** 10.3390/nu15153487

**Published:** 2023-08-07

**Authors:** Silvia Espina, Diego Casas-Deza, Vanesa Bernal-Monterde, María José Domper-Arnal, Sandra García-Mateo, Alberto Lué

**Affiliations:** 1 Gastroenterology Department, Miguel Servet University Hospital, 50009 Zaragoza, Spain; silespina@gmail.com (S.E.); diegocasas8@gmail.com (D.C.-D.); vbernal@salud.aragon.es (V.B.-M.); 2 Adipocyte and Fat Biology Laboratory (AdipoFat), Translational Research Unit, Miguel Servet University Hospital, 50009 Zaragoza, Spain; 3 Instituto de Investigación Sanitaria (IIS) Aragon, 50009 Zaragoza, Spain; mariajoseda_7@hotmail.com (M.J.D.-A.); sgarciamateo7@gmail.com (S.G.-M.); 4 Gastroenterology Department, Hospital Clínico Universitario Lozano Blesa, 50009 Zaragoza, Spain

**Keywords:** malnutrition, sarcopenia, frailty, cirrhosis, alcohol-related liver disease, metabolic dysfunction-associated fatty liver disease, liver transplantation

## Abstract

Liver diseases are the major predisposing conditions for the development of malnutrition, sarcopenia, and frailty. Recently, the mechanism of the onset of these complications has been better established. Regardless of the etiology of the underlying liver disease, the clinical manifestations are common. The main consequences are impaired dietary intake, altered macro- and micronutrient metabolism, energy metabolism disturbances, an increase in energy expenditure, nutrient malabsorption, sarcopenia, frailty, and osteopathy. These complications have direct effects on clinical outcomes, survival, and quality of life. The nutritional status should be assessed systematically and periodically during follow-up in these patients. Maintaining and preserving an adequate nutritional status is crucial and should be a mainstay of treatment. Although general nutritional interventions have been established, special considerations are needed in specific settings such as decompensated cirrhosis, alcohol-related liver disease, and metabolic-dysfunction-associated fatty liver disease. In this review, we summarize the physiopathology and factors that impact the nutritional status of liver disease. We review how to assess malnutrition and sarcopenia and how to prevent and manage these complications in this setting.

## 1. Introduction

Liver cirrhosis is the final stage of chronic liver disease (CLD) and is a condition that predisposes people to the development of malnutrition, sarcopenia, and frailty [1]. Malnutrition is a clinical syndrome that develops from an imbalance, deficiency, or excess of nutrients that leads to an alteration in body composition, resulting in a decrease in physical and mental function [2]. In patients with cirrhosis and malnutrition, there is typically a decline in skeletal muscle tissue (sarcopenia) and a reduction in adipose tissue (adipopenia) [3]. As liver dysfunction progresses in cirrhosis, the prevalence of malnutrition and sarcopenia increases [4,5] and are associated with worse clinical outcomes, including survival and quality of life, and a higher incidence of medical complications, such as ascites, hepatic encephalopathy and increased risk of infections [6]. Furthermore, the dysregulation of the gut microbiome that occurs in cirrhosis may accelerate liver fibrogenesis and cirrhosis-related complications [7].

For this reason, it is advisable for all patients with cirrhosis, especially those with decompensated cirrhosis, to undergo a prompt nutritional screening [8].

Regardless of the etiology of the underlying liver disease, general recommendations have been established in order to identify, prevent, and avoid malnutrition and its complications [2,9,10,11,12,13].

In this review, we summarize the physiopathology of the consequences of nutritional status in liver diseases, which include impaired dietary intake, altered macro- and micronutrient metabolism, energy metabolism disturbances, an increase in energy expenditure, nutrient malabsorption, sarcopenia, frailty, and osteopathy [5]. We evaluate a nutritional assessment in these patients and general nutritional intervention recommendations. Finally, we review particular aspects of nutritional support in specific settings, such as metabolic dysfunction-associated fatty liver disease (MAFLD), alcohol-related liver disease (ALD), liver transplantation (LT), and other infrequent etiologies.

## 2. Consequences of Liver Disease for Nutritional Status

Liver cirrhosis is the consequence of a process of inflammation, destruction, and regeneration of liver parenchyma occurring in any chronic liver disease. The natural history of cirrhosis begins with a compensated phase, with or without portal hypertension, and is followed by a decompensated phase marked by the emergence of major complications, such as ascites, esophageal variceal bleeding, or encephalopathy [14].

Liver cirrhosis is a catabolic condition. The degree of metabolic response depends on the stage of the disease and determines the catabolic rate. Thus, as liver dysfunction progresses, the catabolic rate is higher [15]. Protein–energy malnutrition in cirrhosis arises from a combination of multiple factors, primarily linked to decreased caloric intake, alterations in metabolism, and malabsorption [8,15]. In this section, we summarize the main nutritional factors or consequences involved in the development of malnutrition and sarcopenia in cirrhosis, as well as the mechanisms that explain their appearance in liver disease (Table 1).

### 2.1. Impaired Dietary Intake

The main factor involved in malnutrition is reduced oral intake. Hyporexia or decreased appetite is the main mechanism involved in the reduction in dietary intake in patients with cirrhosis. Hyporexia has been attributed to imbalances between orexigenic and anorexigenic hormones, mainly a decreased cholecystokinin and leptin clearance, and an increase in inflammatory cytokines, such as tumor necrosis factor-alpha (TNF-α) [15]. Other factors causing reduced caloric intake in cirrhosis are early satiety as a result of gastric compression from ascites or abdominal distension due to altered intestinal motility [8,15]. Portal hypertension also contributes to reduced dietary intake by impairing gastric relaxation and motility [15]. Alterations in the oral flora, the use of antibiotics, dry mouth, or zinc or magnesium deficiency can also cause dysgeusia [11]. Furthermore, the other factors implicated in impaired dietary intake are low-salt unpalatability diets in patients with ascites, alcohol abuse, dietary restrictions as a consequence of extended hospitalization periods, and cognitive impairment due to hepatic encephalopathy [8,15,16].

### 2.2. Altered Macro- and Micronutrient Metabolism

#### 2.2.1. Macronutrients Metabolism

Glucose metabolism: It is common for individuals with liver cirrhosis to develop insulin resistance since 60–80% present with impaired glucose tolerance and 10–15% have overt diabetes. Hepatogenous diabetes (HD) is a state of impaired glucose regulation characterized mainly by peripheral insulin resistance in tissues such as skeletal muscle or adipose tissue, while the uptake of glucose in the liver is normal or enhanced [15]. The proposed mechanisms for insulin resistance in cirrhosis are neurohormonal changes, endotoxemia, liver inflammation, altered muscle mass and composition, altered gut microbiota, and permeability [17]. Chronic hyperglycemia causes toxic injury to the pancreatic cells. The accumulation of advanced glycation end-products that are normally cleared by the liver accelerates this process by producing oxidative stress in β-cells. In addition, low-grade systemic hypoxia generated by cirrhosis contributes to the further impairment of the β-cell function. Pancreatic β-cell dysfunction in patients with cirrhosis marks the transition from impaired glucose tolerance to HD [18].

Protein metabolism: Liver cirrhosis is a hypercatabolic state that needs a higher protein intake than usual. The main factors involved in the decrease in muscle mass are the metabolic changes that appear in cirrhosis and alterations in protein turnover [15]. In cirrhosis, gluconeogenesis is increased, and amino acids are the primary source via proteolysis in the skeletal muscle, which generates both branched-chain amino acids (BCAAs) and aromatic amino acids (AAAs) [19,20]. Only BCAAs are catabolized in the skeletal muscle, resulting in lower plasma BCAA levels. Conversely, AAAs are primarily metabolized in the liver, and their plasma levels are increased due to both hepatocellular dysfunction and portosystemic shunting [19]. As the state of liver dysfunction progresses, the plasma BCAA levels decrease [21,22]. A negative correlation has been noted between plasma BCAA levels and the severity of hepatic encephalopathy [23].

Lipid metabolism: Patients with cirrhosis exhibit increased lipolysis, lipid oxidation, and ketogenesis [24].

#### 2.2.2. Micronutrients Metabolism

Vitamin deficiencies in cirrhosis are secondary to diminished reserves due to liver dysfunction, inadequate dietary intake, and nutrient malabsorption [9]. Depending on the etiology, individuals with CLD are at risk of depletion of fat-soluble vitamins, water-soluble vitamins, or minerals. For example, deficiencies of folate (B9), thiamine (B1), zinc, magnesium, selenium, vitamin D, and vitamin E are the most widely recognized deficiencies in patients with alcoholic liver disease [25], while deficiencies in fat-soluble vitamins predominate in patients with chronic cholestatic liver diseases [26]. Vitamin D deficiency (<20 ng/mL) is associated with dysfunction of muscle contractions in the general population and may play a role in the development and progression of frailty in patients with cirrhosis [27]. Patients with vitamin D deficiency can also develop alterations in bone metabolism, such as a significant decrease in bone mineral density (BMD) at the total hip and femoral neck and an increased risk of fractures [28,29]. Severe vitamin D deficiency (<10 ng/mL) is significantly associated with a poor prognosis and with complications of portal hypertension in cirrhosis [28,30]. Zinc deficiency has been linked to the pathogenesis of hepatic encephalopathy, the development of liver fibrosis, and an increased risk of liver carcinogenesis, frailty, and sarcopenia in cirrhosis [31]. Magnesium deficiency occurs due to the malabsorption of magnesium in the small intestine, is exacerbated by diuretic use, and is correlated with diminished muscle strength and reduced cognitive ability in adults with cirrhosis [32]. Elevated manganese levels in patients with cirrhosis have been related to selective manganese accumulation in the basal ganglia, resulting in extrapyramidal and neuropsychiatric symptoms [33]. Specific evidence regarding the advantageous impact of micronutrients and vitamin supplementation in cirrhotic patients is not available. However, confirmed or clinically suspected deficiencies should be addressed via appropriate treatment [9]. Table 2 summarizes the most important micronutrient imbalances in CLD.

### 2.3. Energy Metabolism Disturbances

In liver cirrhosis, hepatocyte dysfunction leads to altered macronutrient metabolism or “accelerated starvation” characterized by diminished hepatic glycogen synthesis and storage, resulting in reduced glycogen stores during the postprandial state [19] and an increased consumption of ketoacids for ammonia metabolism [36]. As a result, muscle catabolism and the use of amino acids for gluconeogenesis are increased [19], which promotes mitochondrial dysfunction [36]. Reduced cellular amino acid concentrations activate responses, including increased skeletal muscle autophagy [19]. The factors that also contribute to stimulating gluconeogenesis in cirrhosis are the proinflammatory state as well as overactivation of the sympathetic nervous system (SMS), which leads to increased levels of epinephrine and norepinephrine and SNS nerve conduction [36]. Additionally, hyperammonemia in cirrhosis activates the expression of myostatin in skeletal muscle, leading to the inhibition of protein synthesis and the activation of autophagy [37]. Other factors that activate protein synthesis and that are decreased in cirrhosis are BCAAs, physical exercise, testosterone, and growth hormone [38]. BCAAs stimulate protein synthesis via mTOR (mammalian target of rapamycin) activation [38].

### 2.4. Increase in Energy Expenditure

The total energy expenditure consists of a combination of the resting metabolic rate, also called the “resting energy expenditure” (REE), the physical activity expenditure, and the diet-induced thermogenesis. The REE represents the total number of calories burned when the body is completely at rest [39] and reflects the energy required to maintain physiological processes, representing approximately 60–70% of the total daily energy requirement. The REE is raised to the tune of 120% of the expected value in more than one-third of cirrhotic patients as a result of the hypercatabolic state [40]. The main mechanisms responsible for this state are hypermetabolism, malnutrition, chronic inflammation, and immunosuppression [3,41]. Additionally, ascites are known to cause increased energy expenditure [42].

### 2.5. Nutrient Malabsorption

There are several factors that contribute to nutrient malabsorption in liver cirrhosis. Fat malabsorption is common, mainly due to impaired bile acid metabolism and small intestinal bacterial overgrowth (SIBO) [43]. A decrease in bile production secondary to impaired liver function affects the formation of micelles that are necessary for fat digestion and the absorption of fat-soluble vitamins [43]. SIBO is very common in cirrhosis, usually secondary to altered intestinal motility. SIBO occurs when colonic bacteria colonize the small bowel, disrupting digestive enzyme production, microvilli function, intestinal barrier, and gut permeability, leading to impaired nutrient absorption and metabolism [44]. SIBO leads to fat malabsorption via deconjugation of bile acids and to amino acid and disaccharide malabsorption as well [45]. SIBO causes excessive production of ammonia and increases the translocation of bacteria and the absorption of bacterial antigens into the bloodstream, which may promote the development of hepatic inflammation, steatosis, and fibrosis [7,45]. Moreover, several complications and decompensations of cirrhosis, such as overt hepatic encephalopathy or spontaneous bacterial peritonitis, are directly or indirectly associated with gut microbiota dysregulation and a decreased diversity of intestinal bacteria [7,45,46]. Other factors that contribute to nutrient malabsorption are pancreatic insufficiency, portal hypertensive enteropathy, and drug-related malabsorption. Pancreatic insufficiency may also contribute to fat malabsorption and is common in patients with alcoholic cirrhosis [43]. Protein loss due to portal hypertensive enteropathy has been described [43]. Lastly, drug-related malabsorption, resulting from diarrhea caused by medications like antibiotics, lactulose, or diuretics, or interference with fat absorption due to drugs like cholestyramine, may also contribute to nutrient malabsorption [44].

### 2.6. Sarcopenia and Muscle Function

Sarcopenia is defined as the “syndrome characterized by a generalized loss of muscle mass, strength and function” [2]. Patients with cirrhosis and malnutrition often have sarcopenia. Like malnutrition, sarcopenia is more prevalent as liver dysfunction progresses, and the prevalence of sarcopenia is higher in patients with decompensated cirrhosis [4,47]. Sarcopenia is linked to a diminished quality of life and a higher risk of cirrhosis-related complications, such as infections, hepatic encephalopathy, and ascites [6]. Furthermore, sarcopenia is an independent predictor of mortality [6].

**Figure 1:**
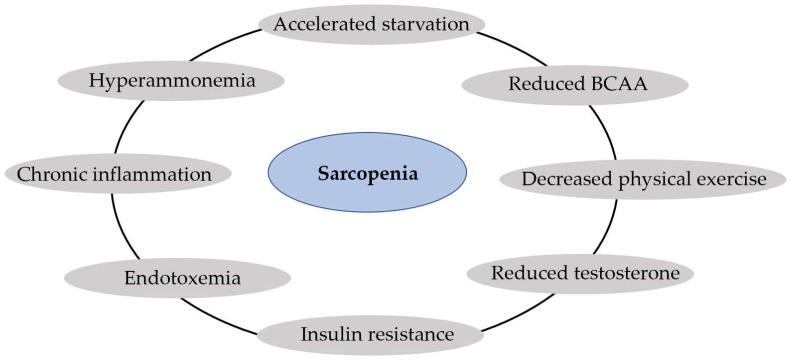
Main factors of sarcopenia in liver cirrhosis.

There are several mediators between the liver–muscle axis, and among them, the most important is hyperammonemia; other mediators are chronic inflammation, endotoxemia from gut translocation, insulin resistance, reduced testosterone, and decreased physical exercise [48]. Hyperammonemia is present in cirrhosis as a consequence of liver cell dysfunction and portosystemic shunt [49]. Hyperammonemia activates in the skeletal muscle the expression of myostatin, a member of the transforming growth factor beta (TGF-β) superfamily produced by myocytes [37]. Myostatin seems to be the mediator of hyperammonemia in the inhibition of protein synthesis and the activation of autophagy, being the connection between liver dysfunction and sarcopenia [37]. Reduced levels of BCAAs due to hypercatabolism, low testosterone levels, and decreased physical exercise are factors that reduce protein synthesis in cirrhosis [38]. In addition, endotoxemia resulting from hepatocellular and immune dysfunction and portosystemic shunt may contribute to impaired protein synthesis and potentially active autophagy via tumor necrosis factor-alpha (TNFα)-dependent and potentially TNF-independent pathways [19], such as reducing micronutrients derived from gut-microbiota (butyrate, acetate) [50]. Figure 1 summarizes the main factors that contribute to sarcopenia in liver cirrhosis.

### 2.7. Metabolic Osteopathy

Osteoporosis is a bone disease characterized by a loss of bone mass and quality that leads to fragility fractures [51]. The diagnosis of osteoporosis is based on the BMD, which is generally measured using dual-energy X-ray absorptiometry (DXA). Osteopenia is a clinical term used to describe a decrease in BMD [51]. According to the World Health Organization, osteoporosis is considered when the BMD is 2.5 standard deviations below the young average value (T-score < 2.5) and osteopenia when the T-score is between −1 and −2.5 [52]. Severe or “established” osteoporosis refers to individuals who meet the densitometric criteria and have one or more fragility fractures [52]. Osteomalacia resulting from poor bone mineralization is uncommon in cirrhosis and is only present in patients with persistent vitamin D deficiency, with severe and long-lasting cholestasis and intestinal malabsorption [9].

Osteoporosis and osteopenia are common complications of CLD, with approximate prevalence rates of 12–55% higher than in healthy people [51]. In CLD, bone loss refers to a reduction in bone formation coupled with an increase in bone resorption. CLD-induced bone fragility depends on the etiology, duration, and stage of liver disease [53]. Osteoporosis is more prevalent in cholestatic chronic liver disease than in other liver diseases, occurring in up to one-quarter of patients [54]. Alcoholic liver disease, hemochromatosis, and MAFLD are associated with reduced BMD levels, and patients with chronic hepatitis C virus are at risk of osteoporosis [51]. In the end-stage of CLD or decompensated cirrhosis, the prevalence of osteoporosis is higher and is detected in up to 38% of patients [55]. The pathogenesis of CLD-induced bone loss is multifactorial. Nutritional, hormonal, metabolic, genetic, and inflammatory factors play a significant role in the pathogenesis of osteoporosis [9]. The presence of one or several factors depends on the etiology of the CLD. The primary cause of metabolic osteopathy in cholestatic liver disease is attributed to deficiencies of vitamin K and D; in hemochromatosis, there is associated hypogonadism; in MAFLD, viral hepatitis, and alcoholic liver disease, the increase in proinflammatory cytokine production such as in TNFα represents the pathophysiological mechanism via the stimulation of osteoclastogenesis [51].

## 3. Nutritional Screening and Assessment of Patients with Chronic Liver Disease

### 3.1. Nutritional Screening and Risk of Malnutrition

All patients with cirrhosis, and in particular patients with decompensated cirrhosis, are recommended to undergo a prompt nutritional screen [8]. There are several nutritional screening tools for individuals with cirrhosis, each with its strengths and limitations. The NRS-2002 and MUST are validated tools used to screen hospitalized patients for their risk of malnutrition and are recommended by the ESPEN (European Society for Clinical Nutrition and Metabolism) in hospitalized cirrhotic patients [56]. The Subjective Global Assessment (SGA) uses data collected during a clinical evaluation to determine the nutritional status without recourse to objective measurements [57]. The SGA has good interobserver reproducibility and is associated with various clinical and prognostic variables of liver transplantation. However, the agreement of SGA with other methods of assessment of nutritional status is low, and it underestimates the prevalence of muscle loss in liver disease patients [9]. The RFH-GA (Royal Free Hospital Global Assessment) is a variation of the SGA for determining the nutritional status of patients with cirrhosis. It is a reproducible tool and correlates with other measures of body composition [57]. However, the time required for this tool and the need for trained personnel for consistent results limits its use [8]. In 2006, a new tool emerged to screen for malnutrition, specifically in the cirrhotic population, the Royal Free Hospital Nutrition Prioritizing Tool (RFH-NPT) [57]. The RFH-NPT uses simple clinical questions that take less than 3 min to complete and can be used by non-specialist staff [8]. It classifies patients into low, medium, or high risk for malnutrition [8]. The RFH-NPT seems to be more sensitive than the NRS-2002 in identifying patients at risk for malnutrition [58], and it may be a useful predictor of disease progression and outcomes for patients with cirrhosis [59]. Actually, the RFH-NPT is recommended by the ESPEN guidelines as the best available tool for malnutrition screening in liver disease [56], and it has replaced the use of the SGA and RFH-GA questionnaire to screen for malnutrition in cirrhosis. The liver disease undernutrition screening tool (LDUST) uses six patient-directed questions to screen for malnutrition in cirrhosis [60]. Its limitation is that it is entirely dependent on the patient’s subjective judgment and has a low negative predictive value [61]. As with the RFH-NPT, it requires further validation [8]. The EASL (European Association for the Study of the Liver) recommends using the RFH-NPT or LDUST questionnaires to screen for malnutrition in cirrhosis [9]. The MNA-SF (Mini Nutritional Assessment Short Form) may also have a role in the screening of malnutrition in patients with cirrhosis, although it requires further validation [62]. Finally, for patients on surgery waiting lists, the Controlling Nutritional Status (CONUT) tool has an important ability to predict complications and post-surgical mortality [63]. Table 3 describes the most frequently used tools to screen for malnutrition in liver cirrhosis.

**Figure 2:**
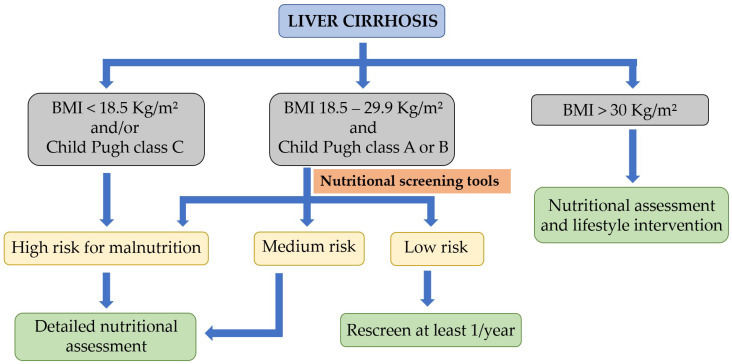
Algorithm for nutritional screening and assessment in liver cirrhosis.

The EASL, in its latest clinical practice guidelines on nutritional assessment and management in chronic liver disease patients [9], recommends that individuals with BMI scores of 18.5–29.9 kg/m^2^ and a Child–Pugh class A or B classification should undergo nutritional screening using one of the liver disease-specific malnutrition screening tools (RFH-NPT or LDUST). On the other hand, patients with a BMI of <18.5 kg/m^2^ or those with a Child–Pugh class C classification or decompensated cirrhosis are considered at higher risk for malnutrition and should undergo a detailed nutritional assessment [8,9]. Figure 2 represents the algorithm for nutritional screening and assessment in liver cirrhosis.

### 3.2. Nutritional Assessment of Malnutrition

A detailed nutritional assessment should be carried out by an expert when any of the recommended tools for nutritional screening classify the patient as medium or high risk for malnutrition. The utilization of nutritional assessment tools will aid in confirming the diagnosis and etiology of malnutrition, assessing its severity, identifying repercussions and deficits, and determining potential interventions to be implemented [64,65].

**Figure 3:**
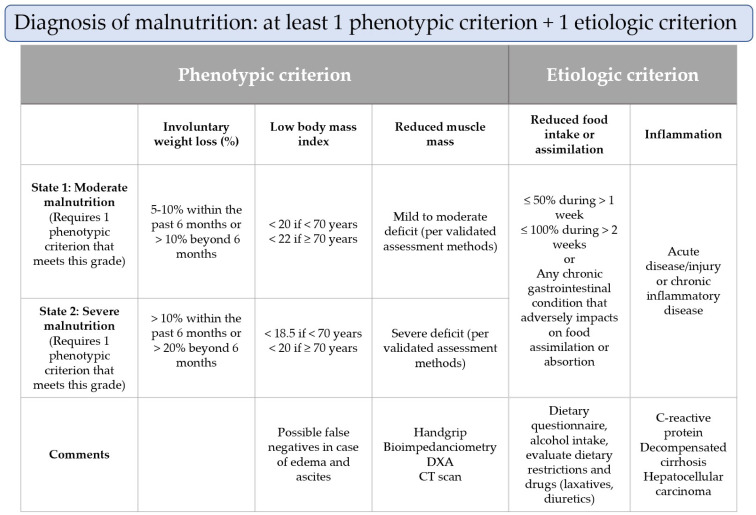
Diagnosis of malnutrition and severity grading based on GLIM criteria.

New diagnostic criteria to confirm a diagnosis of malnutrition were published in 2019 by the Global Leadership Initiative on Malnutrition, called the GLIM criteria [65], with the involvement of leading global nutrition societies. As can be seen in Figure 3, for the diagnosis of malnutrition, the GLIM recommends the combination of at least one phenotypic criterion and one etiologic criterion. The phenotypic criteria include non-volitional weight loss, a low body mass index (BMI), and reduced muscle mass. The etiologic criteria include reduced food intake or assimilation and disease burden or an inflammatory condition. The severity of the malnutrition (moderate or severe) is established based on the phenotypic criteria. According to the GLIM criteria, malnutrition is present in 38.1% and severe malnutrition in 22% of patients with cirrhosis [62]. Guided by the GLIM criteria, we will now examine the specific aspects of each nutritional assessment in patients with liver cirrhosis.

#### 3.2.1. Assessment of Reduced Intake

A dietary intake evaluation requires skilled personnel and relies on patient recall and cooperation [9]. A detailed dietary intake assessment should include food, fluids, supplements, the number of meals and their timing, the number of calories, and the quality and quantity of one’s protein intake [9]. Several tools have been created to assess food intake, including the Food Records (FR), the Food Frequency Questionnaire (FFQ), and the 24-h Recall (24hR) [66]. Today, it is unknown which tools can be considered the most accurate due to the limitations related to misreporting, technical reproducibility, and resource availability [66]. The EASL recommends FRs as the preferred method for assessing food intake in patients with cirrhosis [9]. FRs enable individuals to record their food and drink intake at the time of consumption. Although the minimum time required to record reliable data on food consumption is 3 days, shorter FRs are more commonly used, and patients can complete the diary on non-consecutive days. The FFQ evaluates the frequency of consumption of pre-listed foods and beverages over a specific period (usually weeks). Trained researchers analyze the diary [67]. The 24hR consists of an interview conducted by a trained investigator about all foods and beverages consumed during the preceding day [66].

The dietary interview should also include barriers to eating, such as nausea, vomiting, diarrhea, or constipation, as well as alcohol intake or drug consumption [9]. The EASL recommends asking the patient for the symptom section of the abridged scored patient-generated Subjective Global Assessment [68]. Furthermore, patients should be inquired about any changes in their relative food intake and, if so, the extent of the change and over what period of time [9].

#### 3.2.2. Weight Loss and Body Mass Index

Most nutritional assessment tools include BMI or unintentional weight loss. In cases of fluid retention, it is recommended to subtract a percentage of weight according to the severity of ascites (mild 5%, moderate 10%, severe 15%) and an additional 5% if there is bilateral foot edema [69]. However, the GLIM group warns that patients with liver cirrhosis and obesity or overweight would need to lose substantial weight before receiving a low BMI designation [65]. The GLIM criteria do not require that this item be met, which allows a diagnosis of malnutrition even with a normal BMI or without current weight loss [65].

#### 3.2.3. Muscle Mass and Body Composition

The assessment of muscle mass and body composition is essential since sarcopenia is an important component of malnutrition. There are several nutritional assessment tools that measure body composition, such as anthropometry, a bioelectrical impedance analysis (BIA), DXA, ultrasonography, computed tomography (CT scan), and magnetic resonance imaging (MRI) [8]. In anthropometry, triceps skin fold and mid-arm muscle circumference (MAMC) values are the most commonly used measurements [70,71]. Although anthropometry is simple and rapid, it has interobserver variability and low accuracy [8]. BIA and DXA measurements are affected by fluid retention, and both methods estimate body composition by assessing fat mass and lean mass [72]. BIA is an inexpensive, portable, and simple method with moderate accuracy, while DXA involves radiation exposure and high costs, but its accuracy is high [8]. A skeletal muscle mass estimation by ultrasound is an inexpensive and radiation-free method with moderate to high accuracy. The gold-standard method for sarcopenia assessment is the quantification of muscle mass using cross-sectional imaging with a CT scan [8,72]. The skeletal muscle index (SMI) (cm^2^/m^2^) is calculated by analyzing the abdominal skeletal muscles at the L3 vertebral level. The cut-off values to define sarcopenia vary in individuals with cirrhosis compared to the general population, being ≤39 cm^2^/m^2^ for women and ≤50 cm^2^/m^2^ for men with cirrhosis [73]. However, this method involves radiation exposure and high costs. MRI is an expensive method without radiation exposure, but there is a lack of cut-off values [8].

Muscle strength may be considered if the muscle mass cannot be assessed. The handgrip strength assessment also estimates muscle functionality. It is a cheap, reliable, and reproducible method, but it does not have defined cut-off points in cirrhosis [74].

#### 3.2.4. Disease Burden or Inflammation

The most difficult criterion to evaluate in individuals with CLD is the presence of an inflammatory condition. The GLIM criteria define disease burden or inflammation as the presence of acute inflammatory diseases, such as major infection, burns, trauma, or closed head injury; or the comorbidities of chronic or recurrent mild to moderate inflammation, such as CLD, malignant disease, chronic obstructive pulmonary disease, congestive heart failure or chronic renal disease; or C-reactive protein levels of >5 mg/L [65,75]. According to the GLIM criteria consensus document, the mere presence of a chronic disease, such as liver cirrhosis, is insufficient to meet these criteria. It is necessary to demonstrate the progression or decompensation of the disease to satisfy the criteria [76]. The indicators of inflammation may include fever, negative nitrogen balance, and elevated resting energy expenditure [65].

In conclusion, screening and diagnosing malnutrition in patients with CLD is a complex process influenced by cirrhosis-specific factors. The changes that occur in body composition make it necessary to adapt the global nutritional assessment to obtain accurate results. Consequently, developing specific tools could represent a significant advancement in nutritional evaluation. Regarding the new GLIM criteria, their validity and prognostic capacity require further validation, although their reduced reliance on BMI makes them potentially promising.

## 4. Nutritional Intervention in Liver Disease

### 4.1. Nutritional Intervention in Patients with Hepatic Cirrhosis

In patients with liver cirrhosis, malnutrition rates of around 20% are expected, rising to 60% in those with decompensated cirrhosis [3,11]. In a relationship with the etiology of cirrhosis, it seems that it does not influence the proportion of malnourished patients or their degree of malnutrition, except in cases of patients with alcoholic cirrhosis who more frequently present severe degrees of malnutrition, mainly due to an unhealthy lifestyle and frequent associated socioeconomic problems [77]. In cirrhotic patients who are malnourished, higher rates of development of ascites, hepatorenal syndrome, hospital admissions, derived costs, and mortality have been demonstrated [11].

A personalized nutrition assessment should be provided to all patients with cirrhosis. A reassessment of the nutritional status should be performed at regular intervals, with more frequent intervals reserved for those meeting the criteria for frailty or sarcopenia at baseline or follow-up. If clinical deterioration or a lack of improvement occurs despite target calorie and protein intakes, additional causes should be considered [11].

In patients with cirrhosis, mainly in decompensated patients, the body composition is profoundly altered due to an increase in fluid volume and a decrease in protein. As already mentioned in this chapter, sarcopenia and vitamin and mineral deficiencies are very frequent in these patients and must be evaluated [78].

#### 4.1.1. Energy Intake: General Recommendations, Enteral Nutrition, and Parenteral Nutrition

Nutritional therapy is part of the treatment of patients with liver cirrhosis. A small retrospective study concluded that carrying out this evaluation, as compared to not doing it, implied a benefit in terms of survival for these patients, and if the nutritional evaluation was multidisciplinary (involving a physician, nurse, pharmacologist, and nutritionist), the results were better than if only a single professional assumed the role in this matter [79].

In patients with liver cirrhosis, the daily intake should be divided into six meals, and a late evening snack is essential [80]. There are previous studies that showed that a late-night carbohydrate snack improved protein metabolism in patients with liver cirrhosis. In recent studies, it has been shown that the composition of the late-night snack is not so important, but the important fact is to shorten the fasting periods of these patients by taking an extra food supplement, something that improves insulin resistance and the tendency for sarcopenia in this group of patients [80,81,82].

Patients with liver cirrhosis should take around 30–35 kcal/kg/day. Patients with stable compensated cirrhosis do not have higher energy requirements than the general population. As the disease progresses, these patients have a clear tendency to reduce their caloric intake, something that is especially detrimental in the stage of decompensated cirrhosis (Table 4) [11,83,84,85].

Physical-activity-based interventions have been shown to improve muscle contractile function and muscle mass as well as cardiopulmonary function and quality of life in patients with cirrhosis [1,86,87,88,89,90,91,92,93,94]. The caveat to the interpretation of these studies in this population is that they have been limited by the small sample size and inclusion of primarily well-compensated patients. There are three general principles to consider when recommending activity-based interventions for patients with cirrhosis: assess for frailty or sarcopenia, recommend a combination of aerobic and resistance exercises, and tailor the recommendations based on the physical assessment and reassessments (Table 4) [1,90,92,95,96,97,98].

Obesity is an independent risk factor for worse clinical outcomes in chronic liver disease [13,99,100]. A multicentric, uncontrolled pilot study enrolled patients with compensated cirrhosis, portal hypertension (hepatic venous pressure gradient (HVPG) scores ≥ 6 mm Hg), and body mass index (BMI) scores ≥ 26 kg/m^2^ in an intensive 16-week lifestyle intervention program (personalized hypocaloric, normoproteic diet and 60 min/week of supervised physical activity). Lifestyle interventions led to significantly decreased body weight (BW) (average, −5.0 ± 4.0 kg; *p* < 0.0001). The HVPG scores also significantly decreased (from 13.9 ± 5.6 to 12.3 ± 5.2 mm Hg; *p* < 0.0001). A ≥10% BW loss was associated with a greater decrease in HVPG (–23.7 ± 19.9% vs. –8.2 ± 16.6%; *p* = 0.024). No episodes of clinical decompensation occurred [90].

Enteral nutrition is not beneficial in hospitalized patients with liver cirrhosis when such a prescription has been carried out systematically. It should be performed when the Kcal/day needs are not reached with oral intake or when oral intake is not possible [79,96]. Enteral nutrition improves liver function and survival in these patients. The use of a nasogastric feeding tube may be necessary, and if so, the presence of esophageal varices is not a formal contraindication, but close monitoring is warranted for signs of rebleeding if an enteric tube is required after the recent banding of esophageal varices. The placement of a percutaneous gastrostomy tube has a higher risk of complications in these patients, mainly due to bleeding from collateral circulation in the abdominal wall and gastric wall and complications secondary to the presence of ascites. The pre-procedure assessment for a percutaneous gastrostomy should incorporate laboratory investigations, including a full blood count (with particular attention to the platelet count) and coagulation tests; the recommended thresholds are a platelet count of >50,000/μL and an INR < 1.5 [1,12,84,101,102,103,104,105]. In patients with ascites grades II–III, a percutaneous gastrostomy tube could be placed after paracentesis only if ascites accumulation can be prevented for a period of 7–10 days after tube insertion in order to allow for tract maturation [105].

Parenteral nutrition should be used when oral and enteral nutrition does not meet the nutritional goals or when the gastrointestinal tract cannot be used (for example, in cases of intestinal obstruction) [106]. Its composition should not vary from that of patients without cirrhosis since varying the composition, for example, by adding more branched-chain amino acids, has not demonstrated superiority over a standard composition in these patients, although there are studies that reveal a clinical benefit in patients with hepatic encephalopathy but without improved survival rates in this subgroup of patients [107,108,109,110]. Special care must be taken for the risk of infection of central feeding catheters in these patients, as this is increased, as well as the risk of secondary sepsis [11].

#### 4.1.2. Protein Intake

Patients with liver cirrhosis and without malnutrition should take around 1.2 g of protein/kg/24 h, and patients with liver cirrhosis and malnutrition y/o sarcopenia (including obesity patients with sarcopenia) should take 1.5 g of protein/kg/24 h [11,111]. In patients who are critically ill with cirrhosis, a higher protein target of 1.2–2.0 g/kg/24 h is recommended [1]. To achieve correct muscle recovery, a correct protein intake must be combined according to the needs of the patients with adequate physical exercise. The ideal physical exercise range is between 30 and 60 min of a combination of aerobic and anaerobic exercise each day, and always, if possible, according to the characteristics of each patient (Table 4) [1,90,92,95,96,97,98,112].

In cases of digestive intolerance in a diet composed of rich animal proteins, the diet should be supplemented with vegetable proteins, which are better tolerated in general and additionally have a laxative effect and prebiotic action in these patients [9,113,114].

In men with cirrhosis who may be candidates for testosterone therapy, their testosterone levels should be checked at baseline. Testosterone replacement may be considered in select men with low testosterone to improve their muscle mass. The relative contraindications to the use of testosterone include a history of cancer and thrombosis [1,115,116].

Concerning enteral nutrition supplements, in patients with advanced liver cirrhosis, branched-chain amino acids should be prescribed to improve their complication-free survival and quality of life at a dose of 0.25 g/kg/day [11]. Three recently published randomized clinical trials have shown that the administration of branched-chain amino acid supplements improves hepatic events related to patient survival, hospital admissions, liver function, and quality of life. In these studies, the administration of these amino acids extended their lives by 1–2 years. In patients with previous hepatic encephalopathy, the number of covert hepatic encephalopathy episodes decreased, and their muscle mass generation increased, although they did not show decreased overt hepatic encephalopathy recurrence versus placebo use in this scenario [117,118,119]. In patients with cirrhosis and carcinoma, hepatocellular supplementation with branched-chain amino acids is also recommended, as it improves their response to treatment [120,121].

#### 4.1.3. Vitamins and Minerals

Cirrhotic patients are at risk of deficiencies in water-soluble vitamins, especially B1 (thiamine), as well as fat-soluble vitamins (especially vitamin D) [10,122,123,124,125]. These should be supplemented or added to the treatment of these patients in cases of suspected or confirmed vitamin deficiency [10]. There is a lack of data on the effect of vitamin D supplementation on mortality, quality of life, and serious adverse effects in cirrhosis and CLD, and more evidence is needed [126]. However, the beneficial effects of vitamin D supplementation have been described. In patients with alcohol-related cirrhosis, the supplementation of 1000 IU/day of vitamin D during a period of at least six months led to a decrease in the Child-Pugh score, and a higher level of vitamin D level was related to a lower Child-Pugh score [127]. In a randomized clinical trial including patients with decompensated cirrhosis who received oral native vitamin D3 at a dose of 2000 IU once a day for 12 months, vitamin D supplementation was associated with an increase in skeletal muscle mass index and grip strength [128]. In other randomized clinical trials, including patients with cirrhosis, supplementation with vitamin D for one year improves vitamin D serum levels but does not result in improvement in bone mineral density [129]. The consumption of vitamin A and zinc increases the palatability of food and could conceptually improve the intake of these patients, although supplementation is not used in this context in clinical practice [130]. Zinc deficiency has been correlated in case series studies with the development of hepatic encephalopathy; however, in randomized controlled clinical trials, it has been shown that its administration does not provide any beneficial effect in hepatic encephalopathy, which why it is not recommended to use it systematically in these scenarios [131]. Patients with cirrhosis have an increased risk of refeeding syndrome and B1 deficiency secondary to this (Table 4) [10].

#### 4.1.4. Sodium

Patients with liver cirrhosis, especially those with ascites and related conditions, should be very careful with their sodium intake. A moderate restriction of sodium intake (80–120 mmol/day, corresponding to 4.6–6.9 g of salt) is recommended in patients with moderate, uncomplicated ascites. This is generally equivalent to a no-added-salt diet with the avoidance of pre-prepared meals [10,132]. Despite these recommendations, correct energy and protein intakes should be prioritized, and it has been shown that a strict sodium-free diet of very low palatability can worsen the nutritional status of these patients by reducing their total caloric and protein intakes, something that has a high rate of serious consequences compared to sodium intake in patients with ascitic decompensation and other related complications (Table 4) [133,134].

The development of hyponatremia (serum sodium concentration < 130 mmol/L) in patients with cirrhosis carries an ominous prognosis, and it is a typical situation for patients with advanced cirrhosis and refractory ascites. If it is secondary to diuretics consumption, the treatment must be suspended (especially with serum sodium concentrations < 125 mmol/L). Non-osmotic fluid restriction (1000 mL/day) is helpful in preventing a further decrease in serum sodium levels, but it is seldom effective in improving natremia [132] (Table 4).

**Table 4:** Nutrition and physical activity in patients with liver cirrhosis.

Intake/Uptake	Physical Activity [1,90,92,95,96,97,98,99,100,101,102,103]
Weight-based equations (using ideal body weight) [10,83,84,85]: - Non-obese—target of 30–35 kcal/kg/day - Obese (non-hospitalized, clinically stable): caloric targets stratified by BMI: 25–30 kcal/kg/day (BMI 30–40 kg/m^2^) and 20–25 kcal/kg/day (BMI ≥ 40 kg/m^2^)	Personalized activity prescription: Frequency—Aerobic (4–7 days/week) + Resistance (2–3 days/week) Intensity—Use the talk test (be short of breath but can still speak a full sentence); 3 sets of 10–15 repetitions at a time Time—Start slow and build up: - Aerobic: 150 min per week - Resistance: 1 day per week
Protein intake [1,10]: - Without malnutrition: 1.2 g/kg/24 h - Malnutrition y/o sarcopenia (including obesity patients with sarcopenia): 1.5 g/kg/24 h - Critically ill: 1.2–2.0 g/kg/24 h - All patients: BCAA supplementation 0.25 g/kg/24 h; compensated cirrhosis (to achieve daily protein goals in intolerant to animal protein) and decompensated cirrhosis	—
Frequent, small meals and minimized fasting: daily intake divided into six meals (late evening snack) [80,81,82]	
Micronutrient and vitamin administration if it is suspected or demonstrated [10,122,123,124,125]	
A moderate restriction of sodium intake (80–120 mmol/day) is recommended in patients with moderate, uncomplicated ascites [128]	
Non-osmotic fluid restriction (1000 mL/day) can be useful if serum sodium concentration < 125 mmol/L [128]	
Address barriers to intake (e.g., liberalize sodium restrictions as needed)	

BMI: Body Mass Index; BCAA: branched-chain amino acid.

### 4.2. Nutritional Intervention in Patients with MAFLD

With an increased annual incidence of close to 2 new cases/100 patients/year, the prevalence of non-alcoholic fatty liver disease is between 20 and 30%, nowadays being one of the most frequent causes of cirrhosis and liver transplantation worldwide [135,136].

In order to prevent these deleterious consequences, clinicians have focused their attention on insulin resistance as a pathogenic trigger because patients suffering from MAFLD tend to be obese, diabetics, or have metabolic syndrome.

To achieve this goal, lifestyle interventions have been demonstrated to be the most effective tools, as well as controlling underlying conditions such as hypertension or diabetes across the spectrum of MAFLD patients.

#### 4.2.1. Lifestyle Modifications in MAFLD

Recent data show that regardless of the baseline BMI, weight gain could predict the development of MAFLD even if the BMI remains normal [137]. In this line, it is known that liver fat levels, liver enzymes, and the degree of inflammation and fibrosis could improve from weight reduction via reduced energy intake and increased physical activity [138,139].

Although improvements could already be achieved through reductions of only 5% of initial body weight, the greater the weight loss, the greater the histological improvements, and most researchers concluded that at least 10% of body weight is required for an improved NAFLD activity score (NAS) [140] and fibrosis regression [141]. Accordingly, this 10% weight loss is the target for most lifestyle interventions in the setting of MAFLD.

#### 4.2.2. Roles of Different Micro- and Macronutrients

The dietary pattern to achieve MAFLD resolution has recently been one of the objectives of the research. In line with this, several micro- and macronutrients have been studied.

Related to diets enriched in omega-3 polyunsaturated fatty acids (PUFAs) and their sub-types, eicosapentaenoic acid and docosahexaenoic acid, a recent meta-analysis published by Lu et al. [142] of 10 randomized controlled trials concluded that although omega-3 PUFAs improved GGT and liver fat levels, there was not enough evidence of their efficacy in reducing ALT levels. Moreover, there are conflicting data about the beneficial effects of PUFAs on non-alcoholic steatohepatitis (NASH), fibrosis, or insulin resistance with a tendency for reductions in liver fat when compared to diets enriched with a high saturated fat diet (SFA) [143,144] and with superiority of omega-3 PUFAs over omega-6 when compared within the PUFA subtypes [145].

An additional way to reduce MAFLD development is by drinking coffee (caffeinated or decaffeinated), as it has been demonstrated to have a hepatoprotective effect by decreasing systemic and liver oxidative stress [146]. In line with this, coffee consumption is associated with a lower risk of metabolic syndrome, with an inverse association between consumption and liver fibrosis in MAFLD patients [147].

Related to micronutrients, although a deficit of C and D vitamins showed an association with MAFLD in some studies, the data are controversial, and the vitamins’ role in humans is still unclear [148].

In terms of refined sugars, which tend to be present in the Western diet nowadays, there is strong evidence for the undeniable association between added sugars in foods and drinks and the development of MAFLD. Although it has been demonstrated that a fructose-rich diet increases the hepatocyte production of triglycerides and TNF, the increased gut permeability and consequent endotoxemia may also play a crucial role in this pathogenesis [149,150]. Furthermore, it is important to highlight the importance of the increment in lipid peroxidation and uric acid production in that context [151], which can be associated with insulin resistance and MAFLD development [152].

#### 4.2.3. Dietary Patterns

**Figure 4:**
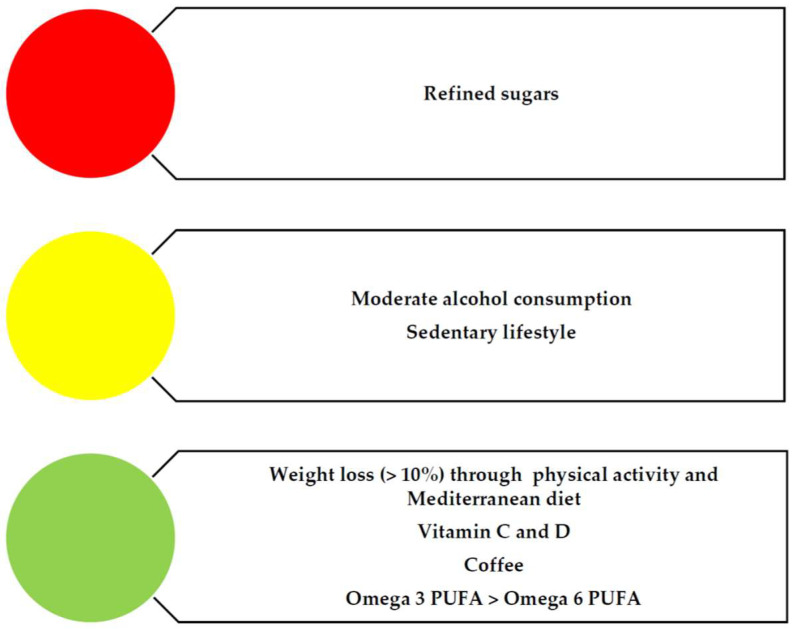
Lifestyle recommendations for patients with MAFLD.

If we focus on dietary models to improve or avoid the development of MAFLD and its complications, the Mediterranean diet (MD) is, without a doubt, the dietary pattern recommended among different scientific societies [153]. The MD diet, based on a high intake of fruits, vegetables, legumes, omega 3 PUFAs, and fish with a low intake of red meat and sweets, has been demonstrated to reduce cardiovascular risk as a primary prevention strategy, as well as reducing diabetes, two crucial comorbidities associated with steatotic liver disease patients [154]. It is unclear if these patients should avoid alcohol intake with the MD diet since any alcohol intake is detrimental to cirrhotic patients due to the greater risk of decompensation and hepatocellular carcinoma, so clinicians tend to recommend that it should be avoided (Figure 4).

Although MD has demonstrated a clear benefit for MAFLD patients, several studies have been conducted in recent years about the potential benefits of intermittent fasting on MAFLD patients.

Intermittent fasting, which uses variable periods of food restriction with specific time frames for eating and with no energy restriction, has demonstrated some potential improvements in biochemical and anthropometric parameters but with inconsistent data due to the very small number of relevant studies in that field [155]; therefore, adherence to a Mediterranean diet remains the most robust dietary recommendation for MAFLD patients.

### 4.3. Nutritional Interventions in Patients with Alcohol-Associated Liver Disease

In recent decades, alcohol-associated liver disease (ALD) has increased in parallel with alcohol consumption. In that line, the rates of steatosis and both alcoholic hepatitis and cirrhosis are currently increasing [156]. With variable rates among different manifestations of ALD, malnutrition is common in those patients, and it has been associated with poor clinical outcomes, with strong correlations between protein–calorie malnutrition and liver disease status [157]. In fact, in a study of 300 outpatients with compensated cirrhosis, the rates of malnutrition varied from 95% in Child–Pugh class C patients to 84 and 46% in classes B and A, respectively [158]. Moreover, a malnourished status could also predict worse outcomes in the long term, as Álvares-Da Silva [159] demonstrated, with higher rates of complications such as uncontrolled ascites, hepatic encephalopathy, hepatorenal syndrome or spontaneous bacterial peritonitis when comparing malnourished patients (66%) with well-nourished ones (12%).

One of the main causes of malnourishment in ALD patients is the decrease in caloric intake, so much so that heavy alcohol consumers derive 50% of their caloric expenditure from alcohol, with an obvious disbalance in proteins, fats, and micronutrients [157]. Moreover, the gut permeability is impaired by alcohol itself, leading to intestinal edema and decreased absorption of micro- and macronutrients [160]. Therefore, changes in gut microbiota are also an undeniable effect of chronic alcohol consumption, which leads to increased firmicute production and, as a result, a reduction in the metabolism of bile acids, which leads to a decrease in fat-soluble vitamins and fats [161].

In that line, patients suffering from ALD are more likely to develop thiamine (B1), B12 vitamin, folate (B9), and vitamin D deficiencies. Lower levels of these micronutrients may cause devasting outcomes such as Wernicke’s encephalopathy, Wernicke–Korsakoff psychosis or wet beriberi (lack of thiamine), macrocytic anemia (B12 and folate deficiency), peripheral neuropathy (B12 deficiency), bone demineralization, fatigue or depression (lack of D vitamin). Investigating and supplementing those potential deficiencies is strongly recommended [162].

As a result of these negative outcomes involving malnourishment, not only alcohol cessation but also nutrition support should be crucial in ALD treatment.

In this way, it is important to highlight that enteral nutrition, apart from the supplementation of micronutrient deficiencies, has shown clear benefits when compared to no intervention or placebo in patients with both cirrhosis and alcoholic hepatitis [163].

#### 4.3.1. General Recommendations

As expected, the main step to start nutritional support in ALD is abstinence from alcohol. Once the main goal is achieved or during the process, a daily protein consumption of 1.5 g/kg body weight and daily caloric intake of at least 35 kcal/kg body weight should be guaranteed.

In line with this, it is important to emphasize its importance to encourage the patient to be adherent to a Mediterranean diet and its characteristic lack of processed foods and added sugars with the avoidance of alcohol consumption. Moreover, the intake times are also crucial in cirrhosis, irrespective of its cause. In this regard, it is very important to ensure intake not only at breakfast but also at bedtime with the aim of avoiding prolonged fasting [9,149,150].

Taking this into account, supplemental nutrition should be guaranteed to those patients who cannot maintain their caloric intake, irrespective of the feeding pattern. In this regard, oral feeding is always preferable to enteral or parenteral, although when oral intake is not available, the ESPEN guidelines recommend enteral feeding or, as a last resort, parenteral feeding, despite the increased risk of non-compliance or infections with nasogastric tubes or parenteral nutrition, respectively [9].

**Figure 5:**
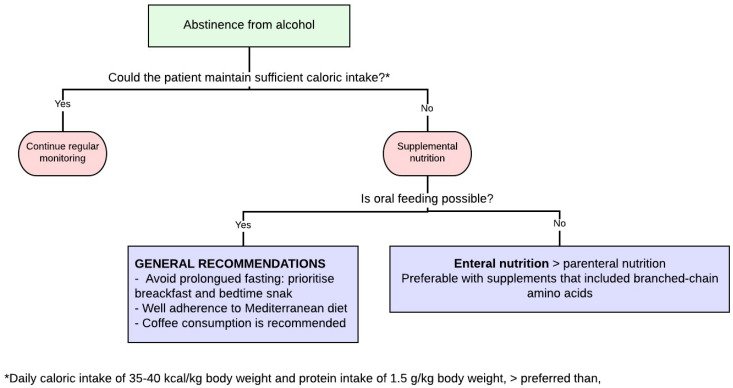
General nutritional recommendations for ALD patients.

Nutritional supplementation, especially with branched-chain amino acids, is associated with lower mortality, with no differences in the mode of supplementation [164] (Figure 5).

#### 4.3.2. Alcoholic Hepatitis

Nutritional support for alcoholic hepatitis has not been in the spotlight among researchers, who tend to focus their attention on other interventions, such as glucocorticoids. However, it has been demonstrated that an oral caloric intake of >3000 kcal/day [165] is strongly associated with a better prognosis in terms of mortality, whereas less than 21.5 kcal/kg is linked with cumulative mortality at six months being independent of MELD as a prognosis factor [166].

Moreover, a meta-analysis of thirteen randomized controlled trials has shown that nutritional supplementation is associated with lower rates of infections and enhanced hepatic encephalopathy [167].

#### 4.3.3. Alcohol-Associated Cirrhosis

Related to alcohol-associated cirrhosis, it has been demonstrated that receiving an energy-dense nutritional supplementation results in lower mortality rates than without it or with a standard oral diet [101].

A systematic review and meta-analysis yielded similar results among 329 ALD patients and 334 controls, with a clear decrease in mortality rates in those ALD patients who received enteral or parenteral nutrition therapy (OR 0.80, 95% CI, 0.64–0.99) [163].

### 4.4. Nutrition-Related Aspects in Liver Transplantation

Liver cirrhosis is the most common indication of liver transplantation (LT) worldwide. The survival rate after LT is currently 70–80% at five years due to the improvements in surgical techniques and the advances in the immunosuppressive regimens that directly affect the outcome of these patients [168]. Malnutrition, sarcopenia and frailty are associated with increased morbidity and mortality after LT and are predictors of unfavorable outcomes [169]. The recipients’ malnutrition was found to be associated with a higher rate of infections, higher surgery-related bleeding rates, and a longer length of stay in the intensive care unit (ICU) [170,171,172,173]. Moreover, in a recent meta-analysis including 3803 patients and evaluating the impact of sarcopenia, it was observed that it is independently associated with post-LT mortality and mortality in waitlist rates, regardless of the Model of End-Stage Liver Disease (MELD) score [174]. Despite this evidence, malnutrition, sarcopenia, and frailty should not be absolute contraindications to LT, even in highly malnourished patients [175]. These results also show the limitation of using the MELD score to prioritize LT in these patients. The MELD score is based on analytical variables (creatinine, INR, and serum bilirubin). It favors the transplantation of the sickest patients and has reduced the mortality of patients on waiting lists [176]. For this reason, many authors have suggested the inclusion of nutritional parameters in the MELD score so as to perfect the prediction of mortality in patients with advanced liver disease. Recently, a new version of the MELD score, the MELD 3.0, including female sex and serum albumin, has been proposed in order to afford more accurate mortality predictions in general than the MELDNa and address determinants of waitlist outcomes [177]. On the other hand, other authors have proposed adding sarcopenia to the MELD score in order to identify and prioritize high-risk patients, particularly in patients who were listed with low priority based on a low MELD score. The presence of sarcopenia is equivalent to adding 10 points to the MELD score in patients included on the wait list for LT [171].

Additionally, MAFLD has become an increasing indication for LT and is currently the second leading cause of LT in the USA, accounting for 21.5% of performed transplants in adults during 2018. Exponential growth has also been seen in Europe, going from 1.2% in 2002 to 8.4% in 2016 [178]. As a result, the prevalence rates of obesity and metabolic syndrome are increasing among LT candidates. Obesity, especially class III (BMI ≥ 40) or higher, represents a major challenge because it is associated with infectious complications, increased mortality, cardiovascular disease, and cancer [179,180]. Moreover, malnutrition and sarcopenia are frequently seen in cirrhotic obese patients. This condition is defined as “sarcopenic obesity”, which has been suggested to be an independent predictor of survival, quality of life, outcome, and responses to stress and surgery [181]. The presence of metabolic complications related to obesity also affects the long-term post-LT outcome. Due to weight gain and immunosuppression, the risks of arterial hypertension, dyslipidemia, and diabetes mellitus incidence significantly increase after LT, with a negative impact on survival [182].

Due to its effect on LT outcomes, screening for malnutrition and sarcopenia is recommended in all cirrhotic patients evaluated for LT, and the need to incorporate a nutrition specialist into multidisciplinary teams is increasingly highlighted [9,168,180,183]. The recommendations to avoid malnutrition are similar to those given in cirrhotic patients who are not on a waitlist for LT (with a total energy intake range of 30–35 kcal/kg/day and a protein intake range of 1.2–1.5 g/kg/day). In obese patients, a lower total energy intake (25 kcal/kg/day) can be given, associated with an increased protein intake (2–2.5 g/kg/day) in order to avoid and prevent sarcopenia [9,168]. Adding oral nutritional supplementation (ONS) has not been demonstrated to be superior to nutritional counseling alone in improving clinical outcomes in these patients [9,56,184]. A meta-analysis of different treatments, such as BCAA, glutamine, and post-LT parenteral nutrition containing fish oil-derived long-chain *n*-3 PUFAs or omega-3 fatty acids, reported overall beneficial effects in terms of decreased infections, decreased length of hospital stay, and improved liver function, although no significant difference in survival was observed [185,186]. Other interventions in LT to ameliorate nutritional support, such as vitamin D supplementation [187] or physical activity, and to improve the muscle mass in LT candidates are recommended [188]. In the early post-LT period, normal food or enteral nutrition should be started within 12–24 h in order to reduce the infection rate [56]. In malnourished patients, particularly if it is anticipated that the patients will be unable to eat for more than two days or the patients cannot maintain an oral intake above 60% of the recommended intake for more than 10 days, aggressive early post-operative nutrition support should be initiated, ideally via the enteral route [56,181].

### 4.5. Nutrition-Related Aspects in Other Etiologies

#### 4.5.1. Nutritional Intervention in Patients with Hereditary Hemochromatosis

Hereditary hemochromatosis (HH) is a genetic disease with an incidence rate of 1 in 250 patients. It is a clinical disease presenting commonly in Northern European males [189]. The disorder occurs as an autosomal recessive disorder in individuals homozygous for the C282Y mutation in the human hemochromatosis (HFE) gene [189]. The disease develops due to iron overload and could affect different organs such as the liver, heart, endocrine system, and musculoskeletal system. In the case of the liver, the disease can lead to cirrhosis in its late stages. However, if the disease is diagnosed and controlled from the onset of cirrhosis, it can be potentially cured. Phlebotomy is the mainstay treatment, which is scheduled according to ferritin levels [190]. There has been no established food and nutrition guidance for diseases characterized by the presence of iron overload (IOL) yet. During treatment with phlebotomies, dietary restrictions are not necessary because the amount of iron removed balances the oral intake of iron. When not receiving phlebotomies, patients with HH must limit their dietary iron intake, avoid supplements containing iron, and also avoid excess vitamin C intake because it may increase their dietary iron absorption [191,192].

Moreover, patients with hereditary hemochromatosis must avoid alcohol consumption. A daily ethanol intake greater than 60 g (5 alcohol units) is associated with an increased rate of cirrhosis [190].

#### 4.5.2. Nutritional Intervention in Patients with Wilson’s disease

Wilson’s disease (WD) is an autosomal recessive disorder caused by mutations of the biliary copper transporter ATP7B. The clinical manifestations of WD depend on the affected system and the degree of copper overload. In more than half of cases, Wilson’s disease starts with liver involvement, which includes hepatic steatosis, chronic hepatitis, cirrhosis, and acute liver failure with hemolysis [193]. Otherwise, the remaining patients have neurological and psychiatric symptoms. The neurological manifestations include resting tremors similar to Parkinson’s disease, rigidity, ataxia, and dystonia. The psychiatric manifestations include depression, worsening school and sports performance, and sexual disinhibition. Wilson’s disease is a rare disorder, affecting 1 out of 30,000 people and clinically manifesting before the age of 40 [193]. The treatment is focused on removing copper from the tissue and excreting it in the urine. For this, chelating agents such as penicillamine and trientine are used. During the penicillamine treatment, the intake of supplements containing vitamin B6 is needed [194,195,196]. In fact, penicillamine is a direct antagonist of vitamin B6, which could negatively affect mitochondrial function via the inhibition of alanine aminotransferase activity and cellular growth [197,198].

Specific dietary guidelines are established for Wilson’s disease. In this regard, patients should avoid foods high in copper, including liver, chocolate, mushrooms, shellfish, and nuts. These dietary restrictions have to be followed strictly, especially in the first year after the diagnosis [198,199]. However, the mainstay of the management of Wilson’s disease remains pharmacological treatment. Indeed, medical treatments are much more effective in managing copper levels than any dietary restrictions.

#### 4.5.3. Nutritional Intervention in Patients with Cholestatic Liver Disease

The cholestatic liver disease represents a unique challenge, with specific challenges in nutrition. Regardless of its etiology, chronic cholestasis can lead to lipid-soluble vitamin and calcium malabsorption, which is strictly related [200]. Fat malabsorption is associated with a decrease in dietary calcium absorption because free fatty acids bind to calcium in the digestive tract, making it unavailable for absorption. Moreover, calcium malabsorption could be worsened by vitamin D deficiency, which is activated to 25-hydroxyvitamin D in the liver. All of these factors mean that most patients with cholestatic disease associated with liver impairment have osteoporosis, which results in significant morbidity [26,200].

On the other hand, calcium malabsorption results in an increase in free oxalate that is easily absorbed and predisposes sufferers to kidney stones.

A deficit of other lipid-soluble vitamins, such as vitamins K, A, and E, is usually present in patients with cholestasis and correlates with bilirubin levels [200]. Oral supplementation is necessary, especially if steatorrhea and malabsorption are present. The oral supplements and tube-feeding products should be high in energy and protein but low in fat. Oral calcium supplements are also recommended, and if administered with 1,25-dihydroxyvitamin D and calcitonin, they can slow the progression of bone disease linked to cholestasis [201]. In cases of severe cholestasis, if fat supplementation is needed, formulas containing medium-chain triglyceride (MCT) oils can be used because they do not need bile acids to be absorbed.

### 4.6. Probiotics as Emerging Treatments for Liver Disease

In recent years, probiotics have emerged as effective treatments for cirrhosis and its complications [202]. Particularly, their role in hepatic encephalopathy treatments has been widely demonstrated.

A recent meta-analysis [203,204,205] of randomized controlled trials demonstrated that probiotics are effective in the treatment of minimal and overt encephalopathy. In the setting of minimal encephalopathy, the effectiveness of probiotics is similar to lactulose, rifaximin, and L-ornithin. When administered together with lactulose, the treatment’s effectiveness does not change. Although probiotics have no effect on mortality, the use of probiotics is safe, and no significant side effects have been described. However, when probiotics are administered for a long time, cases of spontaneous bacterial peritonitis and fatal endocarditis have been reported [202].

Aside from hepatic encephalopathy, in a recent study, probiotics have been demonstrated to improve cognitive functions and increase gait speed in cirrhotic patients while not significantly affecting the risk of falling or the hand grip muscular strength [206].

Moreover, the effect of probiotics on dysbiosis and inflammatory and disease severity markers has been recently demonstrated.

Supplementation with probiotics could directly affect the microbiome composition, ameliorating the dysbiosis of cirrhotic patients. For example, the administration of Lactobacillus GG for a duration of 8 weeks led to an increase in the proportion of beneficial bacteria (Lachnospiracea and Clostridia XIV) and a decrease in the proportion of harmful ones (Enterobacteriaceae). Moreover, this was accompanied by decreases in endotoxemia and systemic inflammation [207]. However, these results have not been observed in other studies using different probiotic strains.

Additionally, the beneficial effect of probiotics seems to be stronger when they are administered in combination. The data regarding VSL#3, which contains eight bacterial strains, seem to confirm this hypothesis. The strains included in the mixture are Lactobacillus acidophilus, Lactobacillus bulgaricus, Lactobacillus casei, Lactobacillus plantarum, Bifidobacteriium brevis, Bifidobacteriium infantis, Bifidobacteriium longum, and Streptococcus salivarius ssp. thermophilus. The administration of VSL#3 in cirrhotic patients has been demonstrated to decrease the Child and MELD scores and serum levels of inflammatory markers (IL-1b, IL-6, TNF-α) and ammonia. Moreover, treatments with this probiotic have direct beneficial effects on hemodynamic markers, such as the hepatic venous pressure gradient, cardiac output, and heart rate, and decrease serum levels of aldosterone, renin, and brain natriuretic peptide [208,209].

In conclusion, probiotics are emerging as potential treatments in patients with cirrhosis and liver disease. Their effectiveness in patients with minimal or overt hepatic encephalopathy has been demonstrated. Some strains seem to have a direct and beneficial effect on dysbiosis and the microbiome composition; however, the results in this setting are controversial [202].

## 5. Conclusions

In conclusion, nutrition in patients with liver disease is crucial for the improvement in clinical outcomes, symptoms, and quality of life and the correction of the consequences on the nutritional status.

In recent years, the physiopathology of the nutritional consequences of liver disease on nutritional status and the impacts on clinical outcomes has been better established. The importance of a systematic assessment of the nutritional status and the incorporation of nutritional interventions has become more and more evident.

Regardless of the etiology of the underlying disease, the physiopathology and clinical consequences are common. Tools to assess and evaluate malnutrition and its complications in all patients are available and easily accessible. Finally, recommendations for nutritional interventions have been established for all patients and for specific settings.

Finally, nutritional assessments and support for liver diseases should be part of clinical practice in order to achieve better outcomes and improve patients’ quality of life.

## Figures and Tables

**Table 1:** Consequences of chronic liver disease for nutritional status.

Nutritional Consequence [Ref.]	Mechanisms in Chronic Liver Disease
1. Impaired dietary intake [8,15,16]	Hyporexia, early satiety, impaired gastric motility, dysgeusia, restrictive diets, and alcohol abuse
2. Altered macro- and micronutrient metabolism [9,17,18,19,20,21,22,23,24,25,26,27,28,29,30,31,32,33,34,35]	Impaired glucose tolerance with insulin resistance and β-cell dysfunction; higher protein intake secondary to hypermetabolism; increased lipolysis and lipid oxidation. Vitamin deficiencies are secondary to malabsorption and diminished reserves
3. Energy metabolism disturbances [19,36,37,38]	Hypermetabolic state causes a reduced hepatic glycogen synthesis and storage and an increase in gluconeogenesis
4. Increase in energy expenditure [3,39,40,41,42]	Hypermetabolism, malnutrition, chronic inflammation and immunosuppression
5. Nutrient malabsorption [43,44,45,46]	Impaired bile acid metabolism, portal hypertensive enteropathy, gut microbiome dysregulation, and small intestinal bacterial overgrowth
6. Sarcopenia and muscle function [19,38,47,48,49,50]	Accelerated starvation, hyperammonemia, endotoxemia, reduced levels of testosterone and branched-chain amino acids, decreased physical exercise
7. Metabolic osteopathy [9,51,52,53,54,55]	Nutritional, hormonal, metabolic, genetic and inflammatory factors

**Table 2:** Micronutrient imbalances in chronic liver diseases.

Micronutrient	Status in CLD	Imbalance-Associated Liver Disease	Liver- and Muscle-Related Consequences
Fat-soluble vitamins [27,28,30]			
A (retinol)	Deficiency	Cirrhosis, MAFLD	Fibrosis development, progression of MAFLD disease
D	Deficiency	Cirrhosis	Liver dysfunction, bone density decreased, progression of frailty, and poor prognosis
E	Deficiency	Alcoholic or cholestatic liver disease, cirrhosis	Increased risk of hepatocellular carcinoma
K	Insufficiency	Cholestatic liver disease	Liver injury, supplementation involved in hepatocellular carcinoma reduction
Water-soluble vitamins [1,25,26,28,34]			
B1	Deficiency	Alcoholic liver disease	Neurologic dysfunction (Wernicke encephalopathy), high-output heart failure
B6	Deficiency	Cirrhosis	Inadequate antioxidant capabilities of the liver
B9	Deficiency	Alcoholic liver disease	Progression of liver disease, muscle weakness
B12	Increase	Cirrhosis, alcoholic liver disease	No symptoms; association with liver fibrosis and liver failure
C	Deficiency	MAFLD	Possible influence on the progression towards MAFLD
Minerals [31,32,33,35]			
Zinc (Zn)	Deficiency	Cirrhosis	Hepatic encephalopathy, liver fibrosis, liver carcinogenesis, myopathy
Magnesium (Mg)	Deficiency	Cirrhosis	Reduced cognitive performance and reduced muscle strength
Manganese (Mn)	Increase	Cirrhosis	Extrapyramidal and neuropsychiatric symptoms
Selenium (Se)	Deficiency	Cirrhosis, chronic hepatitis	Development of hepatocellular carcinoma, increased risk of hepatic encephalopathy, muscle pain
Iron (Fe)	Increase	Alcoholic liver disease, cirrhosis	Liver fibrosis, increased risk of infections

CLD: chronic liver disease; MAFLD: metabolic dysfunction-associated fatty liver disease.

**Table 3:** Most frequently used nutritional screening tools for patients with chronic liver disease.

Screening Tool [Ref]	Variables	Strengths	Limitations
NRS-2002 [56]	BMI Weight loss Food intake Illness severity	Validated in hospitalized patients	Fluid overload can decrease accuracy Low sensitivity in liver cirrhosis
MUST [56]	BMI Weight loss Acute illness and impact on dietary	Validated in hospitalized and outpatients Quick and easy	Fluid overload can decrease accuracy Low sensitivity in liver cirrhosis
SGA [9,56]	BMI Food intake Gastrointestinal symptoms Functional status Comorbidities Physical exam	Good interobserver reproducibility Good association with various clinical and prognostic variables	Underestimates the prevalence of muscle loss in liver disease patients Based on subjective variables
MNA-SF [62]	Weight loss Appetite Mobility Neuropsychological problems BMI Acute illness	Good sensitivity and specificity to screen malnutrition in cirrhosis	Needs more validation
RFH-NPT [8,57,59]	Transplant candidate Fluid overload BMI (in the absence of fluid overload) Weight loss Food intake Acute illness	Liver disease-specific tool Quick and easy Reduces the impact of fluid retention High sensitivity and specificity to screen malnutrition in cirrhosis Useful predictor of disease progression and outcome	Needs more validation
LDUST [60,61]	Food intake Weight loss Body fat loss Muscle mass loss Fluid overload Functional status	Liver disease-specific tool Reduces the impact of fluid retention	Based on subjective variables Low negative predictive value Needs more validation

## Data Availability

No new data were created.
